# Clinical Outcomes of Acute Pyelonephritis in Type 2 Diabetes Mellitus

**DOI:** 10.7759/cureus.74865

**Published:** 2024-11-30

**Authors:** Priyatharicini Anandasekar, Thirumal Valavan Kaliaperumal, Swaminathan Ramasubramanian, Edwin Fernando Mervin

**Affiliations:** 1 Internal Medicine, Stanley Medical College, Chennai, IND; 2 Nephrology, Stanley Medical College, Chennai, IND; 3 Internal Medicine, Government Medical College, Omandurar Government Estate, Chennai, IND

**Keywords:** acute pyelonephritis, emphysematous pyelonephritis, renal dysfunction, type 2 diabetes mellitus, urinary tract infection

## Abstract

Background

Diabetes mellitus (DM) is a prevalent predisposing factor for urinary tract infections (UTIs). Among hospitalized patients with acute pyelonephritis, UTIs are more common, severe, and associated with worse outcomes, particularly in those with type 2 DM. Pyelonephritis in DM patients is more frequently bilateral and linked to greater complications, with 90% of emphysematous pyelonephritis (EMPN) and cystitis cases occurring in diabetic individuals.

Objective

This study aims to evaluate the clinical and microbiological profiles, treatment outcomes, and complications of acute pyelonephritis in patients with type 2 DM.

Methods

A six-month prospective observational study was conducted from March 2016 to August 2016 at Stanley Medical College and Hospital, Chennai, India. A total of 102 hospitalized patients presenting with symptoms suggestive of acute pyelonephritis were included. Data were collected using a clinical questionnaire and analyzed using IBM SPSS Statistics for Windows, Version 20.0 (Released 2011; IBM Corp., Armonk, New York, United States). Patients received culture-sensitive antimicrobial therapy, percutaneous drainage, and renal replacement therapy as required.

Results

Out of 102 patients, 80 (78.4%) had non-emphysematous pyelonephritis (NEMPN), and 22 (21.6%) had EMPN. The mean age was 55.2±10.9 years, with a female predominance of 63 (78.4%) participants. *Escherichia coli* was the most common organism isolated in 70 (68.6%) cases. Renal dysfunction was present in 67 (65.7%) patients, with a higher prevalence in EMPN (22, 100%) compared to NEMPN (45, 56.3%) (p<0.001). Mortality was observed in three (3.9%) patients, and 25 (24.5%) developed de novo or progressive chronic kidney disease.

Conclusion

Early diagnosis and aggressive management of EMPN in type 2 DM patients improve outcomes. Acute pyelonephritis is predominantly observed in women, with fever and loin pain as the most common symptoms. *Escherichia coli* is the primary pathogen, and renal dysfunction is frequent but often reversible. Mortality is low with appropriate management.

## Introduction

Diabetes mellitus (DM) is a chronic metabolic disorder characterized by persistent hyperglycemia resulting from defects in insulin secretion, insulin action, or both. It is a significant global health concern, with its prevalence steadily increasing over the past few decades. The global burden of diabetes is a pressing health crisis, with over 529 million people affected in 2021 and projections exceeding 1.3 billion by 2050 [1]. DM is associated with a myriad of complications, both microvascular and macrovascular, which significantly contribute to morbidity and mortality. Among these complications, infections are notably more frequent and severe in diabetic patients compared to their non-diabetic counterparts. Urinary tract infections (UTIs) are among the most common infections in individuals with DM. The prevalence of UTIs is substantially higher in diabetic patients, and these infections tend to be more severe and recurrent. Acute pyelonephritis (APN), an upper UTI involving the renal parenchyma, is of particular concern in diabetic individuals. The pathophysiology behind the increased susceptibility includes impaired immune function, hyperglycemia-induced tissue damage, and alterations in the urinary tract that facilitate bacterial ascent and colonization [2].

APN is a bacterial kidney infection marked by systemic inflammation and urinary symptoms, often associated with significant morbidity. Emphysematous pyelonephritis (EMPN) is a severe, necrotizing infection of the renal parenchyma and surrounding tissues, characterized by the presence of gas within the renal system. EMPN is predominantly seen in diabetic patients, accounting for approximately 90% of cases. The condition is associated with high morbidity and mortality rates, making early diagnosis and prompt, aggressive management crucial for improving patient outcomes [3]. Despite the recognized association between DM and UTIs, including EMPN, there remains a lack of comprehensive data on the clinical and microbiological profiles, management strategies, and outcomes of APN in type 2 diabetic patients, particularly in the Indian subcontinent [4-8]. The rise of antibiotic resistance poses a significant challenge in the effective management of these infections, necessitating ongoing surveillance and tailored antimicrobial strategies. Diabetic patients often present with atypical symptoms, which can delay diagnosis and treatment, thereby increasing the risk of complications. The high prevalence of comorbidities such as hypertension, cardiovascular diseases, and chronic kidney disease (CKD) in diabetic populations further complicates the clinical management of APN. These comorbidities not only predispose patients to more severe infections but also influence the therapeutic approach and outcomes. There is also a knowledge gap regarding the optimal management protocols for EMPN in diabetic patients. While surgical intervention has traditionally been the mainstay of treatment, recent advancements suggest that conservative management with antibiotics and percutaneous drainage may be equally effective in certain cases. However, the decision-making process remains complex, necessitating a clear understanding of the factors that predict successful outcomes [9].

This study aims to investigate the clinical characteristics, microbiological findings, and treatment outcomes of APN in patients with type 2 DM, with a specific focus on EMPN as a subset.

## Materials and methods

Study design and setting

This study is a six-month prospective observational study conducted from March 2016 to August 2016 at Stanley Medical College and Hospital, Chennai, India. Approval to conduct the study was obtained from the Institutional Ethics Committee of Stanley Medical College (approval number: EC/NEW/INST/2022/TN/0102).

Participants

The study included 102 adult patients diagnosed with type 2 DM who were hospitalized with symptoms suggestive of APN. Inclusion criteria encompassed adult patients (aged ≥18 years) with a confirmed diagnosis of type 2 DM, presenting with clinical signs and symptoms indicative of APN, such as fever, loin pain, and urinary symptoms. Exclusion criteria encompassed patients with CKD stage 3 or higher (estimated glomerular filtration rate (eGFR) <60 mL/min/1.73 m²) or significant proteinuria, immunocompromising conditions, pregnancy, or urinary tract anatomical abnormalities predisposing to infections.

Data collection

Data were systematically collected using a structured clinical questionnaire, encompassing demographic details (age, gender, residence), comorbidities (hypertension, cardiovascular disease, chronic liver disease, cerebrovascular disease, hypothyroidism, malignancy, etc.), lifestyle factors (tobacco and alcohol use), and baseline renal function (eGFR). Clinical manifestations, including fever, loin pain, lower urinary tract symptoms, pyuria, hematuria, oliguria, and altered sensorium, were documented. Laboratory investigations included complete blood count (CBC) to assess for thrombocytopenia and leukocytosis, serum electrolytes to evaluate for hyponatremia, blood urea, and serum creatinine levels to assess renal function. Urine cultures were obtained to identify causative pathogens and their antibiotic sensitivities. Imaging studies, primarily contrast-enhanced computed tomography (CECT) of the abdomen and pelvis, were performed to classify the severity of pyelonephritis and identify complications such as abscess formation or obstruction.

Management protocol

Patients were managed based on clinical protocols tailored to their severity of infection and the presence of complications. Initial management included the administration of empiric broad-spectrum antibiotics, later adjusted according to culture and sensitivity results. In cases of EMPN or abscess formation, percutaneous drainage was performed. Renal replacement therapy, including hemodialysis and intermittent peritoneal dialysis, was initiated in patients presenting with acute kidney injury necessitating such interventions. Double J (DJ) stenting was employed in cases of urinary obstruction to facilitate drainage.

Outcome measures

The primary outcomes assessed included clinical recovery, defined by the resolution of symptoms and normalization of laboratory parameters, development of complications such as de novo CKD or progression of existing CKD, mortality within 90 days of diagnosis, and dialysis dependency post-discharge. Secondary outcomes encompassed the recurrence of APN within the study period.

Statistical analysis

All collected data were compiled and entered into a master chart. Statistical analysis was performed using IBM SPSS Statistics for Windows, Version 20.0 (Released 2011; IBM Corp., Armonk, New York, United States). Descriptive statistics (mean, standard deviation, frequencies, and percentages) were utilized to summarize patient characteristics and clinical parameters. Comparative analyses between non-emphysematous pyelonephritis (NEMPN) and EMPN groups were conducted using independent sample t-tests for continuous variables and chi-squared tests for categorical variables. A p-value of <0.05 was considered statistically significant. Multivariate logistic regression was employed to identify independent risk factors associated with adverse outcomes, with the Hosmer and Lemeshow test used to assess the goodness of fit for the model.

## Results

A total of 102 patients with APN were included in the study, comprising 80 (78.4%) with NEMPN and 22 (21.6%) with EMPN. The study population showed a strong female predominance (80, 78.4%) with a female-to-male ratio of 3.6:1. The mean age was 55.2±10.9 years, with most patients (64, 62.7%) falling in the 51-70-year age group, followed by 31 (30.4%) in the 30-50-year age group and only seven (6.9%) above 70 years. The EMPN group was slightly older (57.8±10.3 years) compared to the NEMPN group (54.5±10.9 years). Most patients resided in rural areas (54, 52.9%), though the EMPN group showed a higher proportion of urban residents (12 (54.5%) vs. 36 (45%) in NEMPN) (Table 1).

**Table 1:** Patient characteristics EMPN: emphysematous pyelonephritis; NEMPN: non-emphysematous pyelonephritis; SD: standard deviation; APN: acute pyelonephritis; CKD: chronic kidney disease

Parameter	NEMPN (n=80)	EMPN (n=22)	Total (n=102)
Age (years, mean±SD)	54.5±10.9	57.8±10.3	55.2±10.9
Gender			
Male	17 (21.3%)	5 (22.7%)	22 (21.6%)
Female	63 (78.7%)	17 (77.3%)	80 (78.4%)
Female-to-male ratio	3.7:1	3.4:1	3.6:1
Residence			
Urban	36 (45%)	12 (54.5%)	48 (47.1%)
Rural	44 (55%)	10 (45.5%)	54 (52.9%)
Comorbidities			
Hypertension	27 (33.7%)	8 (36.4%)	35 (34.3%)
Cardiovascular disease	13 (16.3%)	4 (18.2%)	17 (16.7%)
Chronic liver disease	6 (7.5%)	3 (13.6%)	9 (8.8%)
Cerebrovascular disease	5 (6.3%)	2 (9.1%)	7 (6.9%)
Hypothyroidism	5 (6.3%)	1 (4.5%)	6 (5.9%)
Malignancy	3 (3.8%)	1 (4.5%)	4 (3.9%)
Others	7 (8.8%)	3 (13.6%)	10 (9.8%)
Lifestyle factors			
Tobacco use	29 (36.3%)	7 (31.8%)	36 (35.3%)
Alcohol	23 (28.8%)	5 (22.7%)	28 (27.5%)
Baseline CKD	19 (23.8%)	9 (40.1%)	28 (27.5%)
Recurrence of APN	14 (17.5%)	5 (22.7%)	19 (18.6%)
Poor glycemic control	41 (51.3%)	17 (77.3%)	58 (56.9%)

Comorbidities and risk factors

Hypertension was the most prevalent comorbidity (35, 34.3%), followed by cardiovascular disease (17, 16.7%), chronic liver disease (9, 8.8%), and cerebrovascular disease (7, 6.9%). Baseline CKD was present in 28 (27.5%) patients, with a notably higher prevalence in the EMPN group (9 (40.1%) vs. 19 (23.8%) in NEMPN). Poor glycemic control was observed in 58 (56.9%) patients, significantly higher in the EMPN group (17, 77.3%) compared to NEMPN (41, 51.3%) (p<0.001). Lifestyle factors included tobacco use (36, 35.3%) and alcohol consumption (28, 27.5%). Recurrent APN was documented in 19 (18.6%) cases, with a higher frequency in EMPN patients (5 (22.7%) vs. 14 (17.5%) in NEMPN) (Table 1).

Clinical presentation and disease classification

Fever was the predominant presenting symptom (90, 88.2%), followed by loin pain (87, 85.3%). Lower urinary tract symptoms were significantly more common in the EMPN group (14 (63.6%) vs. 35 (43.8%) in NEMPN; p=0.021). Laboratory findings revealed significant differences between groups, with hyponatremia being more prevalent in EMPN (9 (40.1%) vs. 13 (16.3%); p<0.001) and higher rates of leukocytosis in EMPN (21 (90.5%) vs. 55 (69.1%); p=0.048). Among EMPN cases, the disease severity was classified according to established criteria, with Class 2 being the most common presentation (13, 59.1%), followed by Class 4 (6, 27.3%), Class 3A (2, 9.1%), and Class 1 (1, 4.5%) (Table 2). The distribution of renal involvement showed interesting patterns, with right-sided disease predominating (46, 45.1%), followed by bilateral involvement (32, 31.4%) and left-sided disease (24, 23.5%). The EMPN group showed a higher proportion of right-sided involvement (12 (54.5%) vs. 34 (42%) in NEMPN).

**Table 2:** Emphysematous pyelonephritis classification

Class	Number of patients (n)	Percentage (%)
Class 1	1	4.5%
Class 2	13	59.1%
Class 3A	2	9.1%
Class 4	6	27.3%
Total	22	100%

Microbiological profile and antimicrobial resistance

Urine cultures were positive in 90 (88.2%) patients, with *Escherichia coli* being the predominant organism (70, 68.6%). In the EMPN group, *Escherichia coli* accounted for 14 (63.6%) of isolates, compared to 56 (70%) in NEMPN. Notable differences in pathogen distribution included a higher prevalence of *Enterococcus* in EMPN (3 (13.6%) vs. 2 (2.5%) in NEMPN) and *Proteus* (2 (9.1%) vs. 1 (1.3%) in NEMPN). *Candida* infections were observed in five (4.9%) cases, with similar distribution between groups. Culture-negative cases comprised 12 (11.8%) of the total, with a slightly higher proportion in NEMPN (10 (12.5%) vs. 2 (9.1%) in EMPN) (Table 3).

**Table 3:** Microbiological profile of the study population EMPN: emphysematous pyelonephritis; NEMPN: non-emphysematous pyelonephritis

Organism	NEMPN (n=80)	EMPN (n=22)	Total (n=102)
Escherichia coli	56 (70%)	14 (63.6%)	70 (68.6%)
Klebsiella	5 (6.3%)	-	5 (4.9%)
Pseudomonas	1 (1.3%)	-	1 (0.9%)
Proteus	1 (1.3%)	2 (9.1%)	3 (2.9%)
Enterococcus	2 (2.5%)	3 (13.6%)	5 (4.9%)
Acinetobacter	1 (1.3%)	-	1 (0.9%)
Candida	4 (5%)	1 (4.5%)	5 (4.9%)
Culture negative	10 (12.5%)	2 (9.1%)	12 (11.8%)

Complications and interventions

Acute kidney injury emerged as a significant complication, affecting 67 (65.7%) patients, with 22 (100%) involvement in EMPN cases compared to 45 (56.3%) in NEMPN (p<0.001). Renal obstruction was significantly more common in EMPN (7 (31.8%) vs. 7 (8.8%) in NEMPN; p<0.001), necessitating more frequent urological interventions (10 (45.5%) vs. 7 (8.8%) in NEMPN; p<0.001). Renal replacement therapy was required in 18 (17.6%) cases, though the difference between groups did not reach statistical significance.

90-day outcomes and prognostic factors

The overall 90-day mortality rate was four (3.9%), with a marginally higher rate in EMPN (1 (4.5%) vs. 3 (3.8%) in NEMPN; p=0.812). A composite outcome of de novo CKD, CKD progression, or death occurred in 25 (24.5%) patients, significantly higher in the EMPN group (8 (36.4%) vs. 17 (21.3%) in NEMPN; p=0.006). Post-discharge dialysis dependency affected seven (6.9%) patients, with a higher rate in EMPN (2 (9.1%) vs. 5 (6.3%) in NEMPN) (Table 4).

**Table 4:** 90-day outcomes of the study population CKD: chronic kidney disease; EMPN: emphysematous pyelonephritis; NEMPN: non-emphysematous pyelonephritis

Outcome	NEMPN (n=80)	EMPN (n=22)	Total (n=102)
Mortality	3 (3.8%)	1 (4.5%)	4 (3.9%)
CKD de novo or CKD progression or death	17 (21.3%)	8 (36.4%)	25 (24.5%)
Dialysis dependency	5 (6.3%)	2 (9.1%)	7 (6.9%)

Risk factor analysis

Comprehensive multivariate analysis identified baseline CKD as the sole independent predictor of adverse outcomes, with an odds ratio of 174.52 and a 95% confidence interval of 13.02-2339.49 (p<0.001). In this analysis, several factors that exhibited significance in univariate analysis lost their significance after adjustment. These included gender (p=0.236), altered sensorium (p=0.267), vasopressor use (p=0.075), need for renal replacement therapy (p=0.067), and urological intervention (p=0.962). Furthermore, the multivariate model demonstrated excellent calibration, as evidenced by the Hosmer and Lemeshow test, which yielded a p-value of 0.977, indicating reliable risk prediction capabilities. The findings are summarized in Table 5.

**Table 5:** Analysis of risk factors associated with the study outcome CKD: chronic kidney disease; RRT: renal replacement therapy; OR: odds ratio; CI: confidence interval

Risk factor	Outcome	Univariate p	Multivariate p	OR	95% CI
Age	-	0.635	-	-	-
Gender	-	0.002	0.236	-	-
Baseline CKD	-	0.000	0.000	174.52	13.02-2339.49
Glycemic control	-	0.303	-	-	-
Prior documented pyelonephritis	-	0.839	-	-	-
Platelet count	-	0.105	-	-	-
Serum sodium	-	0.339	-	-	-
Emphysematous pyelonephritis	-	0.144	-	-	-
Altered sensorium	-	0.000	0.267	-	-
Vasopressor use	-	0.000	0.075	-	-
Urological intervention	-	0.051	0.962	-	-
Acute kidney injury	-	0.000	0.998	-	-
Need for RRT	-	0.000	0.067	-	-
Hosmer and Lemeshow test	-	-	0.977	-	-

## Discussion

This study analyzed APN in patients with type 2 DM, highlighting its clinical presentations, microbiological profiles, management, and outcomes. EMPN was present in 22 (21.6%) of the 102 cases, with a mean patient age of 55.2±10.9 years and a female predominance of 80 (78.4%) participants. Fever was observed in 90 (88.2%) patients and loin pain in 87 (85.3%) patients, making them the most common symptoms. EMPN cases showed significantly higher lower urinary tract symptoms, with 14 (63.6%) in EMPN versus 35 (43.8%) in NEMPN (p=0.021). Additionally, EMPN cases exhibited more severe laboratory abnormalities, such as hyponatremia in nine (40.1%) patients compared to 13 (16.3%) in NEMPN (p<0.001) and leukocytosis in 21 (90.5%) versus 55 (69.1%) in NEMPN (p=0.048). *Escherichia coli* was the predominant pathogen, isolated in 70 (68.6%) cases, with other Gram-negative bacilli and *Candida* species also identified, especially in EMPN cases. Imaging studies revealed bilateral pyelonephritis in 32 (31.4%) of patients, and renal obstruction was more frequent in EMPN, occurring in seven (31.8%) patients compared to seven (8.8%) in NEMPN (p<0.001). Management strategies included culture-sensitive antibiotics, urological interventions such as DJ stenting in 10 (45.5%) EMPN cases versus seven (8.8%) in NEMPN (p<0.001), and percutaneous drainage, resulting in a low mortality rate of four (3.9%) patients. EMPN was associated with a higher rate of acute kidney injury, affecting all 22 (100%) EMPN patients compared to 45 (56.3%) in NEMPN (p<0.001). Renal dysfunction occurred in 67 (65.7%) of the overall patient population, and 25 (24.5%) experienced progression to CKD. These findings emphasize the increased severity of APN in diabetic patients, underscoring the need for early diagnosis, aggressive management, and careful renal monitoring to prevent complications and long-term kidney damage.

APN is more severe and frequent in type 2 DM due to chronic hyperglycemia impairing immune function, fostering bacterial growth, and promoting structural kidney changes [10-12]. Advanced glycation end products (AGEs) and oxidative stress weaken neutrophil and macrophage activity, reduce renal microcirculation, and delay immune responses. Diabetic nephropathy and autonomic neuropathy further increase susceptibility by causing urinary stasis, retention, and bacterial proliferation. These mechanisms also elevate the risk of severe complications like EMPN [13,14]. Tight glycemic control and early management of complications are crucial to reducing these risks.

Comparing our findings with Kumar et al., who reported a higher prevalence of EMPN and a greater incidence of renal abscesses, our study showed a slightly lower prevalence of EMPN and fewer abscesses [15]. These differences may be attributable to variations in patient populations, diagnostic criteria, and management protocols. However, both studies consistently highlight the significant morbidity associated with EMPN in diabetic patients. Rollino et al. observed similar trends in female predominance and high rates of renal dysfunction in diabetic patients, reinforcing the consistency of our findings within the broader context of existing research [16]. Their study, conducted over an extended period, included a smaller subset of diabetic patients, yet the observed higher incidence of renal dysfunction aligns with our results, underscoring the inherent vulnerability of diabetic individuals to severe renal infections. Kaye et al. evaluated the efficacy of cefepime/enmetazobactam against piperacillin/tazobactam in patients with complicated UTIs and APN [17]. Their findings suggest that the novel cefepime/enmetazobactam combination shows promise in treating resistant Gram-negative infections, highlighting an alternative treatment option that could be beneficial in cases with antimicrobial resistance, a concern increasingly relevant in diabetic populations where recurrent infections are common.

Bhat et al. conducted a prospective observational study at the RML Institute of Medical Sciences and Eras Lucknow Medical College from 2015 to 2018, encompassing 76 diabetic patients with pyelonephritis, of which 15 (26.3%) were diagnosed with EMPN [18]. This prevalence is slightly higher than our study's 22 (21.6%), which may be attributed to differences in patient populations and referral patterns. Similar to our findings, they reported a predominance of *Escherichia coli* as the causative pathogen, reinforcing the critical role of this organism in EMPN among diabetic individuals. Their study highlighted that poor glycemic control, evidenced by elevated glycosylated hemoglobin levels in EMPN patients, aligns with our observation of poor glycemic control being significantly associated with EMPN. Nabi et al. performed a comparative study on 200 type 2 DM patients with upper UTIs, identifying 20 (10%) cases of EMPN [19]. Their findings corroborate our observation that EMPN, although less common than NEMPN, is associated with more severe complications. Nabi et al. emphasized that EMPN patients frequently presented with vomiting, flank pain, altered sensorium, and renal tenderness, which is consistent with our data showing a higher prevalence of lower urinary tract symptoms and altered sensorium in EMPN cases [20]. They identified additional complications such as diabetic ketoacidosis (DKA), hyperglycemic hyperosmolar state (HHS), and multiorgan dysfunction syndrome (MODS) in EMPN patients, underscoring the systemic severity of EMPN compared to NEMPN. The identification of prognostic factors for poor outcomes in EMPN is critical for risk stratification and management. Bhat et al. identified shock and disturbance of consciousness at hospital admission as independent predictors of poor outcomes [18]. These findings align with our multivariate analysis, which identified baseline CKD as the sole independent predictor of adverse outcomes. The convergence of these studies emphasizes the multifactorial nature of prognosis in EMPN, where both pre-existing renal dysfunction and acute clinical presentations significantly influence patient outcomes.

Similarly, Lee et al. focused on pediatric populations, identifying vesicoureteral reflux and age at diagnosis as significant risk factors for renal scarring post-APN [20]. This underscores the critical role of early diagnosis and intervention in preventing long-term renal damage in children, which may translate to more favorable renal outcomes if applied broadly to high-risk groups. Kumar et al. reported that *Escherichia coli* was the predominant pathogen in diabetic patients with pyelonephritis, with both emphysematous and non-emphysematous forms associated with poor outcomes, especially in those with uncontrolled diabetes. This aligns with our study's observations on the influence of poor glycemic control in EMPN cases and the predominance of *Escherichia coli* as the causative organism in diabetic patients, reaffirming the critical role of rigorous management in reducing morbidity and mortality [15].

The study underscores the necessity for heightened clinical vigilance in diabetic patients presenting with symptoms of APN. Early identification of EMPN through imaging and aggressive management protocols, including appropriate antibiotic therapy and timely urological interventions, is paramount in reducing morbidity and mortality. The significant association of baseline CKD with adverse outcomes highlights the need for comprehensive renal assessment and monitoring in diabetic patients with APN. Furthermore, the high prevalence of poor glycemic control in EMPN patients emphasizes the importance of stringent glycemic management as a preventive strategy against severe UTIs. Regular monitoring of blood glucose levels and optimizing diabetic control can potentially reduce the incidence and severity of APN in this population.

The predominance of *Escherichia coli* as the causative agent necessitates empirical antibiotic therapy targeting Gram-negative organisms. However, the rising trend of antibiotic resistance poses a substantial challenge, necessitating regular updates to empirical treatment guidelines based on local antibiograms [21]. In our study, the high rate of culture sensitivity observed suggests that current antibiotic protocols are effective; however, continuous surveillance is essential to detect emerging resistance patterns. The inclusion of antifungal therapy in cases where *Candida* species were isolated highlights the need for a broad-spectrum approach in severe infections. Tailoring antibiotic therapy based on culture and sensitivity results ensures optimal pathogen eradication and minimizes the risk of resistance development.

This study is limited by its single-center design and relatively small sample size, which may affect the generalizability of the findings. Additionally, the absence of a control group of non-diabetic patients limits the ability to directly compare outcomes between diabetic and non-diabetic populations. Future research should address these limitations by conducting multicenter studies with larger, more diverse cohorts and including matched control groups to provide more robust and comprehensive insights. Longer follow-up periods could also be incorporated into future studies to better understand the long-term renal outcomes in diabetic patients with APN. Another limitation is the potential for selection bias, as the study was conducted in a tertiary care center where more severe cases are likely to be referred. This may have led to an overrepresentation of EMPN and severe APN cases, thereby skewing the prevalence rates and outcomes. Further research should explore the molecular mechanisms underlying the increased susceptibility and severity of UTIs in diabetic patients. Additionally, investigating novel therapeutic approaches, such as targeted antimicrobial therapies and immunomodulatory treatments, could enhance management strategies.

Longitudinal studies tracking renal outcomes over extended periods would provide valuable information on the long-term impact of APN in diabetic populations. Research into the role of prophylactic measures, such as regular screening for UTIs and early intervention strategies in diabetic patients, could also contribute to reducing the incidence and severity of APN. Moreover, exploring the impact of comprehensive diabetes management programs on the prevention of severe UTIs and their complications would be beneficial in developing holistic care models for diabetic individuals.

## Conclusions

Early diagnosis of EMPN in patients with type 2 DM is essential for effective management and improved outcomes. APN predominantly affects women, with fever and loin pain being the most common presenting symptoms, and *Escherichia coli* remains the most frequently isolated organism in urine cultures. A substantial proportion of patients develop renal dysfunction, which is often reversible with appropriate treatment. While this study highlights glycemic control and renal function as critical predictors of EMPN outcomes, additional factors such as antibiotic resistance trends and immunological influences, which might significantly impact patient prognosis, warrant further exploration.

It is also important to note that this study did not include a control group of non-diabetic patients, which limits the ability to draw direct comparisons regarding the influence of diabetes on APN severity. This limitation underscores the need for caution in generalizing these findings to populations without diabetes. Despite these considerations, aggressive management with culture-sensitive antibiotics and percutaneous drainage remains pivotal in achieving favorable outcomes, although altered sensorium and shock continue to be associated with poorer prognoses. These findings highlight the necessity for timely, comprehensive intervention tailored to individual patient contexts.
